# Mean Differential Continuous Pulse Method for Accurate Optical Measurements of Light-Emitting Diodes and Laser Diodes 

**DOI:** 10.6028/jres.126.034

**Published:** 2021-11-23

**Authors:** Yuqin Zong, Jeff Hulett, Naomasa Koide, Yoshiki Yamaji, C. Cameron Miller

**Affiliations:** 1 Sensor Science Division, National Institute of Standards and Technology, Gaithersburg, MD 20899, USA; 2 Vektrex Electronic Systems, Inc., San Diego, CA 92121, USA; 3 Nichia Corporation, Anan-Shi, Tokushima 774-8601, Japan

**Keywords:** continuous pulse, differential method, laser diode, LD, LED, light-emitting diode, mean method, UV-LED

## Abstract

Limited sources exist for the application of germicidal ultraviolet (GUV) radiation. Ultraviolet light-emitting diodes (UV-LEDs) have significantly improved in efficiency and are becoming another viable source for GUV. We have developed a mean differential continuous pulse method (M-DCP method) for optical measurements of light-emitting diodes (LEDs) and laser diodes (LDs). The new M-DCP method provides an improvement on measurement uncertainty by one order of magnitude compared to the unpublished differential continuous pulse method (DCP method). The DCP method was already a significant improvement of the continuous pulse method (CP method) commonly used in the LED industry. The new M-DCP method also makes it possible to measure UV-LEDs with high accuracy. Here, we present the DCP method, discuss the potential systematic error sources in it, and present the M-DCP method along with its reduced systematic errors. This paper also presents the results of validation measurement of LEDs using the M-DCP method and common test instruments.

## 1 Introduction

A potential tool in the fight against healthcare-associated infections (HAI) is germicidal ultraviolet (GUV) disinfection. GUV refers to the ultraviolet-C (UV-C) wavelength range of 200 nm to 280 nm. A limited set of GUV radiation sources is available, including low-pressure mercury discharge tubes, excimer lamps, and now ultraviolet light-emitting diodes (UV-LEDs). Measurement methodologies are required for each of these technologies to enable direct comparisons in the effectiveness of disinfection of pathogens.

Light-emitting diodes (LEDs) and laser diodes (LDs) are commonly measured for optical characteristics (*e.g.*, radiant flux, luminous flux, color, and peak wavelength) at a specified junction temperature, *T*_J_, using the direct current method (DC method), single pulse method (SP method), or continuous pulse method (CP method) [1]. Junction temperature *T*_J_ is an ideal reference measurement condition because almost all optical characteristics of LEDs or LDs are highly sensitive to their *T*_J_. However, *T*_J_ rises rapidly from an initial set temperature due to heating of the junction when performing optical and electrical measurements. As the result, there may be a significant difference between the set *T*_J_ and actual *T*_J_ when the LED is measured. This *T*_J_ difference should be eliminated, compensated, or minimized depending on the measurement method used. In the case of the DC method [2], the *T*_J_ difference is eliminated by first determining the forward voltage, *V*_F_, of the LED at the set junction temperature and then feedback-controlling its mount temperature to maintain the predetermined *V*_F_ value during the optical and electrical measurement period. When a SP method is used, the *T*_J_ difference can be compensated by setting the initial LED temperature to a temperature lower than the specified *T*_J_ so that the optical measurement is performed with the average *T*_J_ being the same as the specified *T*_J_. The exact set initial LED temperature needs to be determined by measuring the LED’s *V*_F_ heating curves [1]. In the case of the CP method, when the pulse width is limited to 50 µs, and the duty cycle is limited to 1% to 2%, the *T*_J_ difference can be minimized to be within 3 °C through conduction. Further, the CP method overcomes the measurement difficulty associated with the anomaly in initial *V*_F_ that often exists when measuring UV-LEDs. The anomaly in initial *V*_F_ is a *V*_F_ surge lasting for several milliseconds when a current pulse is applied to an LED. This anomaly in initial *V*_F_ makes it impossible to use *V*_F_ to infer *T*_J_ during pulsed operation, which is required when the DC method or SP method is used.

The CP method requires that the current pulse waveform is rectangular so that the entire optical measurement is performed at the specified constant operating current, *I*_F_. In practice, however, waveform amplitude distortions, such as overshoot, ripples, and tilt, are inevitable and may be significant due to the current source characteristics and measurement system parasitic impedances, primarily those associated with cable shunt capacitance and series inductance. In addition, slow rise and slow fall of the current waveform introduce so-called rise-and-fall measurement error, which can be significant and is difficult to correct [3]. To improve the CP method, a differential continuous pulse method (DCP method) was recently introduced [4], where a continuous pulse with a long pulse width (CP-L) and a continuous pulse with a short pulse width (CP-S) are used for an optical measurement. The optical measurement result using the DCP method is obtained by subtracting the measured optical signal of the CP-S from that of the CP-L. An individual pulse of the CP-S is composed of mainly rise time and fall time resulting from Gibbs phenomenon [5] and other transients. An individual pulse of the CP-L has a plateau period in addition to the rise time and fall time. The resulting DCP signal represents the light output corresponding to the CP-L where each pulse has the plateau time only. Therefore, the DCP method eliminates the following major error sources in the CP method:

(1) Pulse width and frequency imperfections: Pulse width errors of individual pulses are generally consistent for both CP-L and CP-S, and so they subtract out. For typical current sources with hardware-based timing, pulse width error is reduced by two to three orders of magnitude.
(2) Pulse amplitude distortions: Overshoot and settling time distortions are generally confined to the beginning of the individual pulse, so through appropriate choice of pulse width, they can also be eliminated.
(3) Rise and fall inclusion: The rising and falling edges of individual current pulses of CP-L and CP-S are usually identical, and so the optical measurement errors should subtract out. In reality, the falling edge of the pulse in CP-L produces slightly less light due to higher junction temperature, but with pulse width under 50 µs, the error is generally negligible, as such a short pulse minimizes junction heating.
(4) Large dark signal: Most of the error due to the dark signal (the portion corresponding to the off time of the CP-S) is also removed by the subtraction.

The measurement uncertainty of the DCP method is limited by the remaining errors in pulse amplitude, such as tilt or sag, error in pulse width, and error in pulse frequency. The magnitudes of these errors may be significant depending on the accuracy of the current source (*e.g.*, its jitter) and the measurement setup. To address this issue, we developed a mean differential continuous pulse method (M-DCP method) that eliminates the error resulting from the current pulse amplitude, pulse width, and pulse frequency by measuring the mean currents of the CP-L and CP-S. This mean method was first used to determine the duty cycle of a current CP for measurement of LEDs [6].

## 2 Principle of the M-DCP Method

**Fig. 1:**
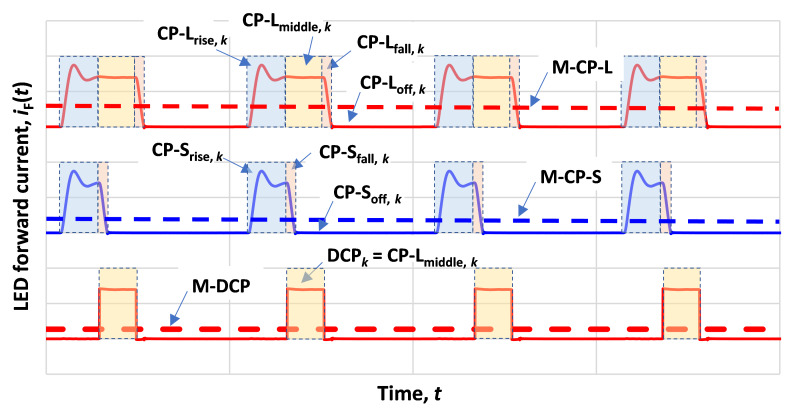
Illustration of the M-DCP method.

The underlying principle of the M-DCP is illustrated in Fig. 1, which depicts a CP-L (the top current waveforms) and a CP-S (the middle current waveforms) during operation of an LED or LD for optical measurements. The CP-L and CP-S have the same pulse repetition rate. The waveform of an individual current pulse, *k*, in the CP-L, denoted as CP-L*_k_*, can be broken down into a rise component, CP-L_rise,_ *_k_*, a fall component, CP-L_fall,_ *_k_*, due to a leading Gibbs phenomenon (ring up) and trailing Gibbs phenomenon (ring down), respectively, and an off component, CP-L_off,_ *_k_*, along with a quasi-steady-state plateau near the middle of the waveform, CP-L_middle,_ *_k_*. The waveform of the corresponding individual current pulse, *k*, in the CP-S, denoted as CP-S*_k_*, has only three components: a rise component, CP-S_rise,_ *_k_*, a fall component, CP-S_fall,_ *_k_*, and the off component, CP-S_off,_ *_k_*. For pulses generated by typical test equipment, the duration and shape of CP-S_rise,_ *_k_* are close to the same as those of CP-L_rise,_ *_k_*, and the duration and shape of CP-S_fall,_ *_k_* are close to the same as those of CP-L_fall,_ *_k_*.

If the current waveform CP-S*_k_* is subtracted from its corresponding current waveform CP-L*_k_*, where the CP-S_fall,_ *_k_* is shifted so it is aligned with the CP-L_fall,_ *_k_*, then the resulting waveform is equal to a nearly ideal rectangular waveform, DCP*_k_*, which corresponds to the middle portion of the CP-L*_k_*, *i.e.*, CP-L_middle,_ *_k_*. This is illustrated in Fig. 1 (bottom waveforms). This classic DCP method was first introduced in 2020 [4].

The DCP method can eliminate the rise-and-fall measurement error in the CP method. Nevertheless, in typical DCP measurements made using general-purpose commercial pulsed current sources, some residual errors remain after the CP-S waveforms are subtracted from their corresponding CP-L waveforms. For example, pulse width and frequency errors not shared by both CP-L and CP-S remain. In addition, pulse amplitude errors in the middle components of the CP-L carry through to the result. Finally, pulse amplitude differences in the rise and fall components of the CP-L and CP-S partially invalidate the assumption that these two components cancel out.

These residual errors could be directly assessed if the DCP waveform could be measured. Because it is an imaginary waveform, it cannot be measured. However, the mean currents of both CP-L and CP-S, the M-CP-L and M-CP-S shown in Fig. 1, can be measured. The mean current of the imaginary DCP, the M-DCP shown in Fig. 1, is the measured M-CP-L minus the measured M-CP-S. This obtained M-DCP value can then be applied as a correction for the DCP measurement, improving its accuracy.

## 3 M-DCP Method Details

The corresponding waveforms of optical signals may be similar to or rather different from the current waveforms shown in Fig. 1, depending on the speed of the optical detector. However, for a pair of individual long-pulse-width and short-pulse-width current pulses *k*, CP-L*_k_* and CP-S*_k_*, including both the pulse-on time period and the pulse-off time period, the corresponding time-integrated optical signals, $Y_{{\text{CP-L}}_{\text{k}}}$ and $Y_{{\text{CP-S}}_{\text{k}}}$, for an optical quantity that is proportional to the operating current are given by

$Y_{\text{CP-L, k}}=\eta_{\text{CP-L}}\int_{0}^{{\text{t}}_{\text{pp}}}i_{{\text{F, CP-L}}_{\text{k}}}\left(t\right)dt=\eta_{\text{CP-L, rise}}\int_{t\;\in \;rise}i_{{\text{F, CP-L}}_{\text{k}}}\left(t\right)dt+\eta_{\text{CP-L, middle}}\int_{t\;\in \;middle}i_{{\text{F, CP-L}}_{\text{k}}}\left(t\right)dt+\eta_{\text{CP-L, fall}}\int_{t\;\in \;fall}i_{{\text{F, CP-L}}_{\text{k}}}\left(t\right)dt+\eta_{\text{CP-L, off}}\int_{t\;\in \;off}i_{{\text{F, CP-L}}_{\text{k}}}\left(t\right)dt$

(1)

and

$Y_{\text{CP-S, k}}=\eta_{\text{CP-S}}\int_{0}^{{\text{t}}_{\text{pp}}}i_{{\text{F, CP-S}}_{\text{k}}}\left(t\right)dt=\eta_{\text{CP-S, rise}}\int_{t\;\in \;rise}i_{{\text{F, CP-S}}_{\text{k}}}\left(t\right)dt+\eta_{\text{CP-S, fall}}\int_{t\;\in \;fall}i_{{\text{F, CP-S}}_{\text{k}}}\left(t\right)dt+\eta_{\text{CP-S, off}}\int_{t\;\in \;off}i_{{\text{F, CP-S}}_{\text{k}}}\left(t\right)dt$

(2)

where

*k* is an integer index,

*t*_pp_ is the current pulse period for the CP-L and CP-S,

$i_{\text{F, }{\text{CP-L}}_{\text{k}}}(t)$ and $i_{{\text{F, CP-S}}_{\text{k}}}(t)$ are the current waveform functions of CP-L*_k_* and CP-S*_k_*, respectively,

$\int_{t\;\in \;middle}i_{{\text{F, CP-L}}_{\text{k}}}(t)dt$ is the integrated signal within the middle component of CP-L*_k_*,

$\int_{0}^{{\text{t}}_{\text{pp}}}i_{{\text{F, CP-L}}_{\text{k}}}(t)dt$ and $\int_{0}^{{\text{t}}_{\text{pp}}}i_{{\text{F, CP-S}}_{\text{k}}}(t)dt$ are the total (time-integrated) currents of the CP-L*_k_* and CP-S*_k_*,

$\int_{t\;\in \;rise}i_{{\text{F, CP-L}}_{\text{k}}}(t)dt$ and $\int_{t\;\in \;rise}i_{{\text{F, CP-S}}_{\text{k}}}(t)dt$ are the integrated currents within the rise components,

$\int_{t\;\in \;fall}i_{{\text{F, CP-L}}_{\text{k}}}(t)dt$ and $\int_{t\;\in \;fall}i_{{\text{F, CP-S}}_{\text{k}}}(t)dt$ are the integrated currents within the fall components,

$\int_{t\;\in \;off}i_{{\text{F, CP-L}}_{\text{k}}}(t)dt$ and $\int_{t\;\in \;off}i_{{\text{F, CP-S}}_{\text{k}}}(t)dt$ are the integrated currents within pulse-off (dark)

components,

$\eta_{\text{CP-L}}$ and $\eta_{\text{CP-S}}$ are the conversion factors for CP-L*_k_* and CP-S*_k_* over the entire regions of the current waveforms,

$\eta_{\text{CP-L, rise}}$ and $\eta_{\text{CP-S, rise}}$ are the conversion factors for CP-L*_k_* and CP-S*_k_* in the rise regions of the current waveforms,

$\eta_{\text{CP-L, fall}}$ and $\eta_{\text{CP-S, fall}}$ are the conversion factors for CP-L*_k_* and CP-S*_k_* in the fall regions of the current waveforms,

$\eta_{\text{CP-L, off}}$ and $\eta_{\text{CP-S, off}}$ are the conversion factors for CP-L*_k_* and CP-S*_k_* in the off regions of the current waveforms, and

$\eta_{\text{CP-L, middle}}$ is the conversion factor from the integrated current to an integrated optical signal for CP-L*_k_* in the middle region of the current waveform.

Using Eq. (1) and Eq. (2), the differential time-integrated optical signal, $Y_{\text{DCP, k}}\;\left({=Y}_{{\text{CP-L}}_{\text{k}}}-Y_{{\text{CP-S}}_{\text{k}}}\right)$, is given by

$Y_{\text{DCP, k}}=\eta_{\text{CP-L, middle}}\int_{t\;\in \;middle}i_{{\text{F, CP-L}}_{\text{k}}}\left(t\right)dt+\left[\eta_{\text{CP-L, rise}}\int_{t\;\in \;rise}i_{{\text{F, CP-L}}_{\text{k}}}\left(t\right)dt-\eta_{\text{CP-S, rise}}\int_{t\;\in \;rise}i_{{\text{F, CP-S}}_{\text{k}}}\left(t\right)dt\right]+\left[\eta_{\text{CP-L, fall}}\int_{t\;\in \;fall}i_{{\text{F, CP-L}}_{\text{k}}}\left(t\right)dt-\eta_{\text{CP-S, fall}}\int_{t\;\in \;fall}i_{{\text{F, CP-S}}_{\text{k}}}\left(t\right)dt\right]+\left[\eta_{\text{CP-L, off}}\int_{t\;\in \;off}i_{{\text{F, CP-L}}_{\text{k}}}\left(t\right)dt-\eta_{\text{CP-S, off}}\int_{t\;\in \;off}i_{{\text{F, CP-S}}_{\text{k}}}\left(t\right)dt\right]$

(3)

For a metrology-grade pulse current source, the rise characteristics of individual current pulses of CP-L*_k_* and CP-S*_k_* are normally the same; likewise, the rise components of the two corresponding integrated optical signals are normally the same. Thus,

$\left[\eta_{\text{CP-L, rise}}\int_{t\;\in \;rise}i_{{\text{F, CP-L}}_{\text{k}}}\left(t\right)dt=\eta_{\text{CP-S, rise}}\int_{t\;\in \;rise}i_{{\text{F, CP-S}}_{\text{k}}}\left(t\right)dt\right]$

(4)

The fall components of the current pulses of the CP-L*_k_* and CP-S*_k_* are also the same. Nevertheless, due to additional heating that occurs during the current pulse CP-L*_k_*, the fall component of the corresponding optical signal pulse is not exactly the same as the fall component of the optical signal resulting from the current pulse CP-S*_k_*. However, for typical LED measurements that involve pulse widths that differ by 10 µs or less, the junction temperatures, *T*_J_, and phosphor (if present) temperatures are approximately the same during the fall time of the current pulses CP-L*_k_* and CP-S*_k_*, and thus,

$\eta_{\text{CP-L, fall}}\int_{t\;\in \;fall}i_{{\text{F, CP-L}}_{\text{k}}}\left(t\right)dt\approx \eta_{\text{CP-S, fall}}\int_{t\;\in \;fall}i_{{\text{F, CP-S}}_{\text{k}}}\left(t\right)dt$

(5)

The standard uncertainty of this approximation is estimated to be within 0.05% for typical pulse widths of 10 µs for CP-S*_k_* and 20 µs for CP-L*_k_*. Using Eq. (4) and Eq. (5), Eq. (3) becomes

$Y_{\text{DCP, k}}=\eta_{\text{CP-L, middle}}\int_{t\;\in \;middle}i_{{\text{F, CP-L}}_{\text{k}}}\left(t\right)dt+\left[\eta_{\text{CP-L, off}}\int_{t\;\in \;off}i_{{\text{F, CP-L}}_{\text{k}}}\left(t\right)dt-\eta_{\text{CP-S, off}}\int_{t\;\in \;off}i_{{\text{F, CP-S}}_{\text{k}}}\left(t\right)dt\right]$

(6)

Equation (6) indicates that the signal components associated with the rise region and the fall region of the CP-L*_k_* measurement are subtracted out by the corresponding rise signal and fall signal of the CP-S*_k_* measurement. Further, the “dark” signal in the CP-L*_k_* measurement is also subtracted out by the term in the bracket, which is the difference of the two integrated “dark” signals during CP-L*_k_* and CP-S*_k_* measurements. Therefore, the DCP signal for pulse *k*, *Y*_DCP,_ *_k_*, represents only the dark-corrected signal associated with the middle component of the current pulse *k* of the CP-L*_k_*, which corresponds to the flat, relatively undistorted region.

During DCP measurements, optical instruments normally integrate signals over a time period that includes multiple pulses, and the integrated signals are averaged over time. Therefore, the measurement equations for the optical signals for the CP-L and CP-S are given by

$Y_{\text{CP-L, mean }}=\frac{\sum_{k=1}^{n}\left(Y_{{CPL}_{\;k}}\right)}{t_{\text{int}}}$ (7)

and

$Y_{\text{CP-S, mean}}=\frac{\sum_{k=1}^{n}\left(Y_{{CPS}_{k}}\right)}{t_{\text{int}}}$ (8)

where

*Y*_CP-L, mean_ is the measured mean optical signal for the CP-L,

*Y*_CP-S, mean_ is the measured mean optical signal for the CP-S,

*n* is the total number of pulses, which does not need to be set, and

*t*_int_ is the set integration time (often called measurement aperture) of the instrument, which is exactly an integer multiple of the pulse period *t*_pp_ of the CP-L and CP-S.

The corresponding mean optical signal of the DCP is the time-averaged sum of signals of all individual optical pulses, given by

$Y_{\text{DCP, mean}}=\frac{\sum_{k=1}^{n}Y_{DCP,\;k}}{t_{int}}=\frac{\sum_{k=1}^{n}\left(Y_{{CPL}_{k}}-Y_{{CPS}_{k}}\right)}{t_{\text{int}}}=Y_{\text{CP-L, mean}}-Y_{\text{CP-S, mean}}$ (9)

where

*Y*_DCP, mean_ is the mean optical signal of the DCP.

Equation (9) indicates that the DCP optical signal corresponding to measurement of a rectangular CP (the DCP waveform shown at the bottom of Fig. 1) is simply the difference between the measured mean CP-L signal and the measured mean CP-S signal.

Spectroradiometers, photometers, radiometers, and tristimulus colorimeters can all be used for optical measurements using the differential measurement equation in Eq. (9). Note that Eq. (9) applies only to measurement of optical quantities that are proportional to the operating current. Therefore, optical quantities such as peak wavelength or chromaticity coordinates must be obtained from the measured mean DCP spectral signals, *Y*_DCP, mean_.

If the specified condition for the optical measurement has the same amplitude and frequency as those of the DCP, then the mean DCP signal is the properly scaled result. However, in most cases, the equivalent DC (100% duty cycle) measurement is desired, and so Eq. (9) must be scaled to obtain the intended result. For typical DCP measurements, the scaling factor is the reciprocal of the DCP duty cycle, and the programmed duty cycle is taken as being equal to the actual duty cycle.

As discussed above, this equivalence is not always true; for example, pulse period jitter can make the CP-L or CP-S periods differ from each other. In addition, pulse amplitude errors, such as ringing and tilt during the middle portion of a pulse of the CP-L, can affect the result. These residual errors are further reduced using the M-DCP method by first calculating the true mean forward current of the DCP, *I*_F, DCP, mean_, the M-DCP shown in Fig. 1, and then using this calculated value to scale the DCP result. Like the mean differential optical signal, *Y*_DCP, mean_, using Eq. (9), the mean differential forward current, *I*_F, DCP, mean_, can be calculated from the measured mean currents of the CP-L and CP-S,

*I*_F, DCP, mean_ = *I*_F, CP-L, mean_ - *I*_F, CP-S, mean_ (10)

where

*I*_F, CP-L, mean_ is the measured mean forward current of the CP-L, the M-CP-L shown in Fig. 1, and

*I*_F, CP-S, mean_ is the measured mean forward current of the CP-S, the M-CP-S shown in Fig. 1.

Finally, the mean DCP optical signal, *Y*_DCP, mean_, must be converted to the equivalent measurement performed under the corresponding nominal DC current operating condition, *Y*_DC_, given by

$Y_{\text{DC}}=Y_{\text{DCP, mean}}\times \frac{I_{F,\;amplitude}}{I_{F,\text{ DCP, }mean}}$ (11)

where

*I*_F, amplitude_ is the nominal (programmed setpoint) forward current amplitude of CP-L.

By using this forward current ratio in place of the reciprocal of the DCP duty cycle, the conversion using Eq. (11) performs the necessary duty cycle scaling while at the same time correcting for the residual duty cycle and amplitude errors in the DCP measurement. For an LED or LD that is only sensitive to *T*_J_, the result of a M-DCP measurement made in this way will match the result of the DC measurement to within the measurement uncertainty. However, a real-world LED or LD may also be sensitive to some extent to its package temperature and ambient temperature; therefore, a small difference may be observed when comparing the DC result converted from the M-DCP measurement result using Eq. (11) to that directly measured under DC operation at the same *T*_J_.

Using the M-DCP method, the measured result represents the characteristics when the LED junction and entire package have the same temperature, *T*_J_, assuming the heating is negligible. In comparison, when the LED is measured under DC operation, the measured result reflects the characteristics of the LED under a thermal equilibrium condition with a temperature gradient over the package. In the case of a phosphor-converted LED, the phosphor temperature may be tens of degrees Celsius higher than the junction temperature, *T*_J_. Because of the two different thermal conditions, the DC and M-DCP measured results for some phosphor-converted LEDs may differ, even if the junction temperatures are controlled to be the same during both measurements.

When actual DC operation and measurement are desired (*e.g.*, time-consuming gonio-spectroradiometric measurements), the M-DCP method can be used to determine the value of a suitable temperature-sensitive parameter (*e.g.*, luminous flux, radiant flux, illuminance, irradiance, *λ*_P_, or *V*_F_ if no initial transient voltage anomaly effect exists), with the temperature-controlled mount (TCM) set to a specified junction temperature *T*_J_. The TCM can then be adjusted lower to match the chosen parameter measured in the DC condition to that measured using the M-DCP method. This is essentially the reverse of the match used in the validation (see Sec. 6). Once a match is obtained, the junction temperature *T*_J_ under this DC operation is the specified junction temperature *T*_J_. Under this condition, all optical and electrical measurements can be measured at the DC condition.

## 4 Practical Implementation of the M-DCP Method

The mean forward currents, *I*_F, CP-L, mean_ and *I*_F, CP-S, mean_, can be measured with high accuracy using a digital multimeter (DMM) with a dual-slope or multislope integrating analog-to-digital converter [7].^1^1 Certain commercial equipment, instruments, or materials are identified in this paper to specify the experimental study adequately. Such identification does not imply recommendation or endorsement by the National Institute of Standards and Technology, nor does it imply that the materials or equipment identified are necessarily the best available for the purpose. Nearly all bench-top and laboratory DMMs use such an analog-to-digital converter for the direct current (DC) function and DC voltage function. For these functions, the DMM integrates the input signal within a specified integration time window (also called the measurement aperture) and then determines the integration-time-averaged value (mean value).

In principle, the DMM’s measurement of the mean value (DC value) should not be influenced by the large-amplitude components in a CP-L or CP-S if the DMM is not saturated at the peak of the pulse. However, we found some higher resolution DMMs (7 ½ or 8 ½ digits) exhibited a small offset error when measuring CP-L and CP-S signals with amplitude exceeding 30% of the meter range. Nevertheless, when calculating the mean DCP optical signal, *Y*_DCP, mean_, using Eq. (9), (or the mean DCP forward current, *I*_F, DCP, mean_, using Eq. (10)) the two offset errors in the CP-L measurement and CP-S measurement canceled out. It is possible to check for the presence of this error by switching the DMM to a range that is one level higher than required for the maximum pulse amplitude. For DMMs with the offset, the results will differ. For these DMMs, the higher range should be used.

A high-accuracy, high-speed (10 million samples per second or higher) digitizer may also be used for measuring the mean optical signals or mean forward currents of the CP-L and CP-S with a pulse width of 10 µs or longer. However, the cost of such a digitizer is high, and its measurement uncertainty is typically larger than that of a DMM.

For optical measurements, as in Eq. (7) and Eq. (8), the instrument’s integration time, *t*_int_, should be matched to exactly an integer multiple of the pulse period, *t*_pp_, of CP-L and CP-S (*e.g.*, 1 time, 2 times, 30 times, 100 times, …) to obtain a stable measurement result. In such a case, each measurement always includes the same number of pulses. As it is not necessary to begin the optical integration at a specific point in the pulse train, accurate hardware triggering for synchronization between the current source and the optical instrument is not required.

The test circuit should use twisted wires, and the pulsing parameters of the current source should be tuned so that the rise component and fall component in the pulse waveform are minimized.

When testing LEDs or LDs at high current levels (>10% of their rated forward currents), the current pulse width of the CP-L should be as short as possible to minimize heating during the pulse time. LED heating is in most cases insignificant when the current pulse width is less than 20 µs. For LD, shorter pulses may be necessary. The current pulse width of the CP-S is optimally chosen such that it is just long enough to cover the rise component, pulse width error, pulse width jitter, and fall component of the pulse of the CP-L. The minimum pulse width of the CP-S can be determined by analysis if the current source specifications are known or by checking the current waveform of the CP-L with a high-quality current probe and oscilloscope.

**Fig. 2:**
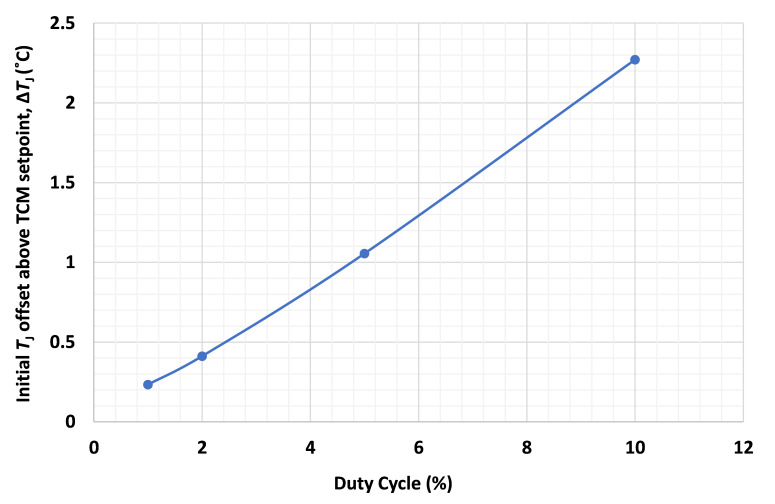
Initial *T*_J_ offset above the TCM setpoint (Δ*T*_J_) *vs*. duty cycle.

The duty cycle must also be kept low to reduce the average heating. Fig. 2 shows the *T*_J_ offset of a white LED mounted on a TCM just before the pulse when it is powered with a CP having a pulse width of 20 µs at its nominal maximum current of 1 A. The initial *T*_J_ is the set TCM temperature, and the initial *T*_J_ offset above the TCM setpoint, Δ*T*_J_, is the difference between this initial *T*_J_ and the *T*_J_ measured just before the individual pulses. As the graph shows, if the duty cycle of the CP does not exceed 2%, then Δ*T*_J_ is below 0.5 °C.

For low-power testing (< 10% of the rated forward current), instead of simply increasing the number of pulses measured during the optical integration time, a higher duty cycle may be used; this provides a greater optical signal, shortening the needed optical integration time, *t*_int_.

In both the DCP and M-DCP methods, the optical instrument must have a low dark signal because the duty cycle of the CP is usually very low, indicating that the instrument measures its own dark signal during most of the measurement time (integration time, *t*_int_). For example, when testing an LED using a CP-S with a duty cycle of 1%, the signal-to-dark ratio will be two orders of magnitude lower compared to that when the LED is measured by the same instrument under DC operation. On the other hand, because of the low duty cycle, the LED temperature may be set and controlled using a passive heat sink and ambient air (instead of using a TCM).

Note that Eq. (10) cannot be used to obtain the mean forward voltage *V*_F_. When an LED is off between two consecutive pulses, the LED voltage level is still significant compared to that when the LED is on during a pulse due to capacitance. Therefore, the LED forward voltage must be measured at the time when a pulse of the CP-L is in the middle region using a trigger-delay-measure scheme. Such a measurement can be performed using a DMM (or a digitizer) that is triggered with a hardware trigger signal from the current source. The DMM should be configured to have a trigger delay time that is equal to the rise time of the pulse of the CP-L and a measurement integration time that matches the optical measurement time, *t*_int_, *i.e.*, the time period of the pulse’s middle region of the CP-L. Precision pulsed current source measure instruments (pulsed SMUs) are also able to make the necessary synchronized measurements using an internal trigger/boxcar averaging scheme.

**Fig. 3:**
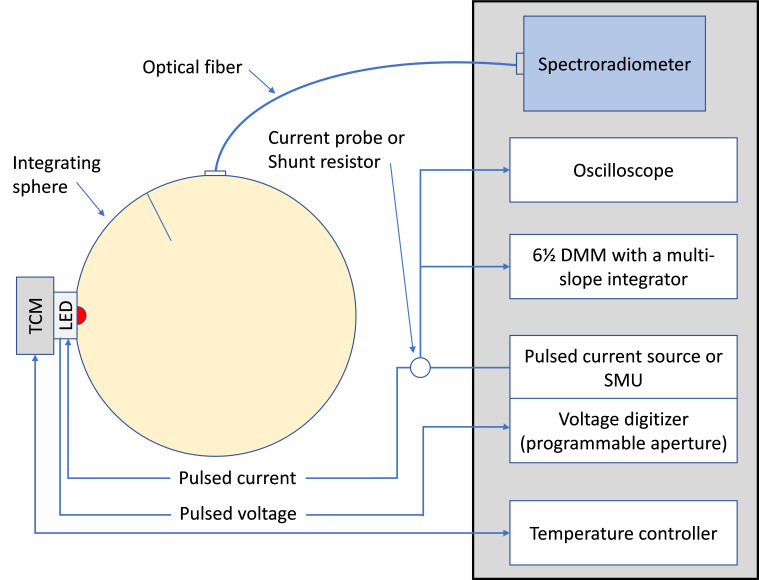
Example measurement setup using the M-DCP method.

Fig. 3 shows an example LED measurement setup that supports the M-DCP method. The LED to be measured is mounted on a TCM. The mean forward current of the LED, *I*_F, DCP, mean_, is measured by a DMM indirectly using the DMM’s DC voltage function and a current probe (or a shunt resistor) that converts the current signal to a voltage signal. The mean forward current of the LED, *I*_F, DCP, mean_, can also be measured directly using the DMM’s DC function.

## 5 M-DCP Method Uncertainty Analysis

The M-DCP method improves upon the DCP method by removing current waveform errors that persist after the subtraction. These include errors due to dynamic variations in the pulse width and frequency

(*i.e.,* jitter), and amplitude errors occurring during the middle component. If the mean current measurements are taken precisely at the same time as the optical measurements, and they encompass enough pulses so that pulse width and frequency variations are accurately measured, then the resulting M-DCP measurement uncertainty is reduced to the error of the current measurement setup. Strictly speaking, this analysis is only valid if the measured optical parameters are perfectly linear with respect to current. For example, radiant flux and luminous flux are quite linear over narrow current ranges, and so M-DCP can provide a substantial correction.

Table 1 shows an uncertainty analysis for a DCP measurement and the corresponding M-DCP measurement made with a commercially available pulsed current source. For the mean current correction, a 6 ½ digit DMM and a precision shunt are assumed. For this example, M-DCP provides improved uncertainty by more than an order of magnitude.

**Table 1:** Uncertainty budgets for typical DCP and M-DCP measurements.

Source of Uncertainty	Relative Standard Uncertainty (%)	
	DCP Method	M-DCP Method
CP-S pulse width, 0.2 µs^a,b,c^	1.00	N/A
CP-L pulse width, 0.2 µs^a,b,c^	0.50	N/A
CP-S pulse width jitter, 0.11 µs	1.10	N/A
CP-L pulse width jitter, 0.11 µs	0.55	N/A
CP-S pulse period, 2 µs^d^	0.20	N/A
CP-L pulse period, 2 µs^d^	0.20	N/A
CP-S pulse period jitter, 2 µs^d^	0.20	N/A
CP-L pulse period jitter, 2 µs^d^	0.20	N/A
Amplitude error during middle component	0.50	N/A
CP-S DMM mean voltage for measuring mean current through a shunt resistor	N/A	0.05
CP-L DMM mean voltage for measuring mean current through a shunt resistor	N/A	0.05
Resistance of shunt resistor	N/A	0.05
Relative combined uncertainty (%)	1.8	0.09
Relative expanded uncertainty (%) (*k* = 2)	**3.6**	**0.17**

a The pulse width and duty cycle are 20 µs and 2% for CP-L, and 10 µs and 1% for CP-S.

b Pulse width imperfections are sometimes similar for two pulses generated by the same current source. For example, if the current source has a +1 µs error at 20 µs, then it may exhibit the same error at 10 µs; therefore, in the DCP subtraction, the two pulse width errors cancel out.

c Value assumes that half of current source pulse width error is common to both CP-S and CP-L.

d Pulse period imperfections may or may not be similar for the first and second measurements. Even if they are similar, the error is not removed by the DCP subtraction.

## 6 Validation of the M-DCP Method

### 6.1 Validation of Mean Current Measurement Using a DMM

To obtain an accurate mean DCP forward current, *I*_F, DCP, mean_ from Eq. (10), it is critical for the DMM to be able to measure the mean currents, *I*_F, CP-L, mean_ and *I*_F, CP-S, mean_, of the low-duty-cycle pulsed current waveforms, or at least obtain measurements with consistent errors that cancel out when the difference is taken. To determine if commonly used DMMs can do this, an experimental M-DCP measurement was set up. A precision pulsed current source was used to generate CPs with pulse widths of 10 µs for CP-S and 20 µs for CP-L and duty cycles of 1% for CP-S and 2% for CP-L. The pulse amplitude was set to be 0.11 A, and the DMM was set up to measure DC voltage using a current probe (with a gain setting of 1 V/A). The measurement range of the DMM was set to be 1 V to simulate the worst measurement condition (*i.e.*, the input signal is only 11% of the instrument range). The integration time of the DMM was set to 0.25 s. In total, 10 measurements were made, and the average of the 10 readings was used. Seven commonly used DMM models (four 6 ½, one 7 ½, two 8 ½) from three different manufacturers were used for this experiment to determine if different models/manufacturers had any difficulty with the measurement.

The results of the experiment are shown in Table 2. The experiment showed that the *I*_F, CP-S, mean_ measurement was lower than the expected value by about 68%, and *I*_F, CP-L, mean_ was lower by 34%. These large errors were due to the bias error of the current probe and likely also due to the pulse width error of the current source. While this error reduced the measured individual *I*_F, CP-L, mean_ and *I*_F, CP-S, mean_ values significantly, the calculated *I*_F, DCP, mean_ value was within 0.9% of the expected value. This remaining 0.9% error was likely a true amplitude error, meaning the amplitude of the current during the flat segment of the pulse of the CP-L was actually 0.9% too high. Each of the seven DMMs was able to correctly determine the *I*_F, DCP, mean_. In fact, the agreement among all DMMs was on the level of 0.1%, which includes the measurement error resulting from stability of the current source. Before this experiment, the reference 8 ½ DMM (DMM Model No. 7) was compared to a high-performance digitizer (0.1% standard uncertainty, 16bits, 20 MHz bandwidth, 20 million samples per second). The agreement of the measured *I*_F, DCP, mean_ values was within 0.1%. Thus, commonly used DMMs are able to make measurements with the required accuracy and consistency.

**Table 2:** Measurement of the mean forward current using seven different models of DMMs.

DMM	Resolution	*I*_F, mean, PS_	*I*_F, mean, PL_	*I*_F, mean, DCP_	Normalized to DMM Model No. 7
DMM Model No. 1	6 ½	3.521E−04	1.464E−03	1.112E−03	0.999
DMM Model No. 2	6 ½	3.492E−04	1.461E−03	1.112E−03	0.999
DMM Model No. 3	6 ½	3.486E−04	1.460E−03	1.111E−03	0.999
DMM Model No. 4	6 ½	3.505E−04	1.465E−03	1.115E−03	1.002
DMM Model No. 5	7 ½	3.455E−04	1.458E−03	1.113E−03	1.000
DMM Model No. 6	8 ½	3.496E−04	1.462E−03	1.112E−03	1.000
DMM Model No. 7	8 ½	3.519E−04	1.465E−03	1.113E−03	1.000

### 6.2 Validation of Optical Measurement

The optical measurement results using the M-DCP method were compared to those under DC operation. Four LEDs were chosen for the test: two white LEDs, one ultraviolet (UV) LED, and one amber LED. The LEDs were mounted to a TCM as shown in Fig. 3 and measured using both the M-DCP method and the DC method. Each LED was first measured under DC with the TCM programmed to 25 °C and the forward current shown in the table. The LED *T*_J_ was allowed to rise to a point dictated by the path’s thermal resistance to the TCM. After the LED thermally stabilized, the radiant flux, *Φ*_e_, was measured using a spectroradiometer with a spectral range from 200 nm to 800 nm, and forward voltage, *V*_F_, was measured using a digitizer. Then, the LED was measured for *Φ*_e_, *V*_F_, and peak wavelength, *λ*_P_, using the M-DCP method by setting the current source to produce a CP-L with a pulse width of 20 µs and duty cycle of 2%, and a CP-S with a pulse width of 10 µs and duty cycle of 1%. Before the M-DCP measurement, the TCM temperature was adjusted so that the measured *V*_F_ matched the *V*_F_ value obtained from the DC measurement. The *V*_F_ was measured during the middle component of a pulse of the CP-L using the same digitizer used for measuring the V_F_ under DC operation. Last, the LED was measured again under DC operation with the peak wavelength, *λ*_P_, matched to the one obtained from the M-DCP measurement. This was done by adjusting the TCM temperature.

The comparison results are shown in Table 3. The differences between the M-DCP and DC methods vary from -0.2% to 4.2%. Both *V*_F_ and *λ*_P_ are sensitive to the operating junction temperature, and thus both should accurately reflect *T*_J_. However, as other research has shown, some LEDs, in particular, some amber LEDs and UV-LEDs, exhibit initial transient voltage anomaly effects that distort the initial *V*_F_ values and make the measured *V*_F_ value too high when the M-DCP method is used [8]. The junction temperature column in Table 3 is in fact the TCM temperature for M-DCP measurement assuming the *T*_J_ rise is negligible.

**Table 3:** Comparison of measurement results using the M-DCP method and DC method.

LED Type	Forward Current, *I*_F_ (A)	Forward Voltage, *V*_F_ (V)	Junction Temperature, *T*_J_ (°C)	Peak Wavelength, *λ*_P_ (nm)	Difference of Radiant Flux, *Φ*_e_ (M-DCP Relative to DC), at the Same *V*_F_	Difference of Radiant Flux, *Φ*_e_ (M-DCP Relative to DC), at the Same *λ*_P_
Cool white LED #1	0.350	3.475	36	452.79	0.0%	−0.3%
Cool white LED #2	1.000	3.525	54	440.37	4.1%	4.2%
UV-LED	0.350	6.402	44	274.43	−1.0%	−0.2%
Amber-color LED	1.000	2.313	72	597.21	−1.6%	2.1%

For the first LED, the cool white LED #1 with color temperature of 10 000 K, the two measured radiant fluxes matched perfectly when *V*_F_ was used as a temperature-sensitive parameter. The flux measurements also matched well when *λ*_P_ was used.

For the second white LED, the cool white LED #2 with color temperature of 6300 K, the measured radiant fluxes did not match. Both the *V*_F_ and the *λ*_P_ results were about 4% higher for M-DCP. The cause of this large difference was due to the high thermal sensitivity of the phosphor. Under DC operation, the LED’s phosphor temperature is tens of degrees Celsius higher than its junction temperature, *T*_J_, which lowers the radiant flux value. In contrast, the agreement with the measured results for the cool white LED #1 was within 0.3%, implying that the conversion efficiency of the phosphor was not sensitive to its temperature in the studied temperature range.

The UV-LED showed a lower flux reading when *V*_F_ was used for matching. This is consistent with a small initial transient voltage anomaly effect that slightly increases the *V*_F_ during the first few hundred microseconds of operation. The voltage offset results in a TCM temperature setting such that the actual *T*_J_ is too high during M-DCP measurements. When *λ*_P_ was used, the flux readings matched within 0.2%.

The last LED, the amber-color LED, might also suffer from an initial transient voltage anomaly effect. Thus, the M-DCP measurements taken when *V*_F_ was used for matching were 1.6% low. When *λ*_P_ was used for matching *T*_J_, the measured radiant flux using the M-DCP method was 2.1% higher, with an unknown cause. Nevertheless, the magnitude of the radiant flux difference corresponded to a difference in *T*_J_ of only 1 °C.

## 7 Summary

A new, practical M-DCP method was developed for optical measurement of LEDs and LDs. The M-DCP method improves upon the measurement uncertainty of the unpublished DCP method by one order of magnitude. The DCP method was already a significant improvement on the CP method commonly used in the LED industry. Experimental results showed that the method’s crucial measurement—determination of the mean current from the pulsed current source—is accurate and simple to perform with commonly available measurement equipment. Tests with various LED types showed that the method produces results comparable with DC operating condition measurements once other factors such as phosphor temperature and initial transient voltage anomaly effect are considered.

UV-LEDs are being developed and used rapidly for air and surface disinfections in healthcare facilities and public areas. High-accuracy optical measurements of the UV-LEDs for such applications are critical due to associated safety and effectiveness issues. The presented new M-DCP method makes it possible to perform such needed measurements. The National Institute of Standards and Technology is developing a new gonio-spectroradiometer facility using the M-DCP method to provide such measurement services for UV-LEDs.
